# Whisker-Mediated Texture Discrimination

**DOI:** 10.1371/journal.pbio.0060220

**Published:** 2008-08-26

**Authors:** Mathew E Diamond, Moritz von Heimendahl, Ehsan Arabzadeh

## Abstract

Rats use their whiskers to rapidly and accurately measure the texture of objects. The authors evaluate recent evidence about how whisker movement across a surface produces texture-specific motion signals, and how the signals are represented by the brain.

Our sense of touch provides information about nearby objects that can affect us in an immediate way. Texture, a central component of touch, is sensed quickly, even before an object is explored to measure its size, shape, or identity. To learn how contact with a surface produces a sensation of texture, many laboratories have examined the whisker system of rodents. Touch sensed through the whiskers in rodents works differently than touch sensed through the fingertips in primates. Touch receptors in the fingertips are distributed in a continuous sheet; this spatial distribution of inputs gives important signals about texture [1]. In contrast, rodents use a set of roughly 30 whiskers on each side of the snout, palpating surfaces through a 5–15 Hz forward-backward motion known as “whisking.” When a whisker's tip or shaft makes contact with a texture, its movement changes; whisker motion signals report to the brain what the whiskers have contacted.

The performance of rats in discriminating textures is astonishing. In the dark, they can extract the identity of a texture based on just one to three touches per whisker and can display accurate judgments of a texture within 100 ms of initial whisker contact [2]. Whisker-mediated texture discrimination has many lessons to teach neuroscientists about sensor mechanisms, central encoding, and the transformation of sensory representations to behavioral output. It is not surprising, then, that whisker touch has become a focus of engineers who look to biology for inspiration in their attempt to endow robots with sense of touch (see, for example, http://www.biotact.org/). This Primer summarizes our current understanding of how whisker motion becomes, for the animal, a texture sensation.

Whisker motion signals are picked up by sensory receptors—the terminations of trigeminal ganglion cells—that convert mechanical energy in the follicle into trains of action potentials. After synaptic relays in the trigeminal nuclei of the brain stem and in the thalamus, signals reach the somatosensory region of the cerebral cortex [3]. The somatosensory cortex contains a set of neuronal populations called “barrels,” each barrel responsible for processing the input from one whisker. Due to their grid-like arrangement, the whiskers can be labeled like cells in a spreadsheet (i.e., A1, C4, E2, etc). Adjacent whiskers project to adjacent barrels, so the barrel field forms an isometric map of the whiskers [4] and assumes the same labeling. Thus, for example, the several thousand neurons in barrel C3 are excited primarily by movement of whisker C3 (and much less by nearby whiskers, like C2 and C4). Neuronal activity within the barrel field is critical to the sensation of texture [2,5].

Though all investigators agree that texture sensation begins with whisker motion, two hypotheses compete to explain which features of whisker motion vary according to texture. The “resonance hypothesis” argues that textures are converted to a spatial code distributed across the whisker pad on the snout. Whisker length increases systematically from the front to the back of the rat's snout [6,7]. Mechanical resonance frequency increases with whisker length, so there is a spatial gradient in frequency tuning of whiskers from the front to the back of the snout [7]. According to the resonance hypothesis, whisker motion across a given texture drives mechanical resonance specifically in those whiskers that possess the resonance frequencies best matching the texture's spatial frequency [8,9]. Thus, the full set of short-to-long whiskers separates textures in the same way that the cochlea—a frequency analyzer par excellence—separates tones. Then, the map-like projection from whiskers to cortex causes each texture to excite a specific subset of barrels. In the resonance hypothesis, the spatial pattern of activity in the barrel cortex encodes the spatial frequency spectrum of the contacted texture.

The “kinetic signature hypothesis” views resonance as an unavoidable consequence of the whisker structure (a tapered elastic beam), but irrelevant for the sensation of texture. This view stresses the conversion of surface shape into precisely timed motion events by individual whiskers [10]. All the whiskers that touch a texture transmit information, and texture identity is encoded by the magnitude and temporal pattern of high and low velocity whisker events [10,11]. This movement profile—the texture's kinetic signature—is determined by surface features like the size of grains and the distance between them. Sensory receptor neurons respond to the most prominent features of the signature—the high velocity jumps over texture grains—and texture-specific firing rates and firing patterns are transmitted to barrel cortex [10].

In a new study published in *PLoS Biology*, Jason Wolfe et al. carried out innovative experiments aimed at selecting between these hypotheses [12]. They trained rats to whisk against sandpapers of different grain size while recording whisker motion with a linear array of optic sensors (Figure 1). When whiskers were not touching a texture but freely moving in air, their motion was continuous and smooth. But moving along the texture, their trajectory was characterized by an irregular, skipping motion: the whisker tip tended to get fixed in place (“stick”), before bending and springing loose (“slip”) only to get stuck again (Figure 2). A slip-stick event was a jump in speed and acceleration; the two quantities covaried.

**Figure 1:**
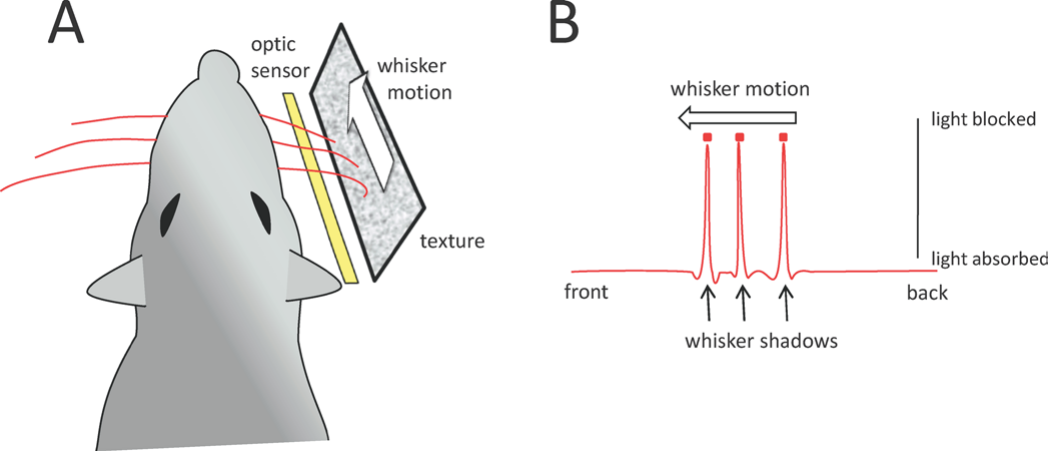
Setup for the Experiment of Wolfe and Colleagues (A) An optic sensor was placed below the textured plate; the rat palpated the texture with its whiskers. (B) The position of each whisker (red square centered at peak of the whisker shadow) was measured at 4 kHz frame rate and ~5 micron spatial resolution. Adapted from [12].

**Figure 2:**
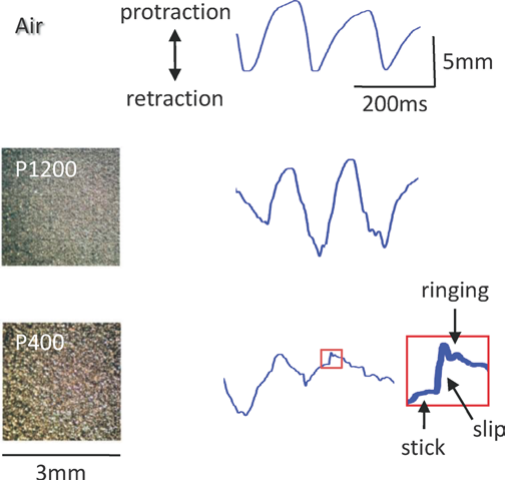
Whisker Trajectories Whisker trajectory measured when the whiskers moved through the air with no texture present (upper trace) and when the whiskers contacted texture P1200 (middle trace) and texture P400 (lower trace). Adapted from [12]. Texture photographs from [10].

How do the two texture coding hypotheses measure up against Wolfe's findings? The rate and magnitude of slip-stick events varied systematically with texture. On coarse textures, there were more high-speed and high-acceleration slip-stick events, while on smooth textures, there were more low-speed and low-acceleration slip-stick events. So the ratio of the number of high to low magnitude events, in single whiskers, gave a remarkably fine “kinetic signature” of the contacted texture. Pairs of textures that behaving rats are known to be able to discriminate [2,13–15] were clearly separable from each other by this measure of whisker motion.

After some of the slip events, the whisker vibrated for a few cycles at its resonance frequency (see also [16]). However, this “ringing” was a characteristic of the whisker, not of the texture. A given whisker resonated equally for all textures, so the presence or absence of resonant vibrations carried no information about texture. Taken together, these findings fit the kinetic signature hypothesis and argue against the resonance hypothesis.

An important question is whether texture encoding by kinetic signatures is specific to Wolfe's experimental set-up or is valid across different conditions. Because whisking is an actively controlled sensory-motor behavior [17,18], its parameters vary from moment to moment. Moreover, a rat may encounter the textured surface to the side of its snout (as in Wolfe's study), in front of the snout [2,13], or on the ground. To find out whether a single encoding mechanism works under all these conditions, we compared the whisker motion from Wolfe with that obtained in our laboratory in a completely different setting [10]. Animals were anesthetized and “electrical whisking” was induced by direct stimulation of the facial nerve, the motor bundle innervating the muscles of the whisker pad. Textures were positioned 7 mm from the snout (about 20 mm in Wolfe's study), and the surface was coplanar with the trajectory of whisker motion (orthogonal in Wolfe et al.). Despite the different conditions, the same kind of whisker movement was found in both studies: each texture generated a kinetic signature, a distinct motion pattern folded into the whisker trajectory. Observing side-by-side traces of whisker velocity obtained from the two studies reveals a striking similarity (Figure 3). Figure 4A makes the comparison in a more quantitative manner. In Wolfe's study, the number of high-magnitude slip-stick events per whisk (red points) increased for progressively coarser textures; by the same token, in our study [10], the equivalent noise level (ENL; blue triangles) increased for progressively coarser textures (ENL is a measure of energy related to the number and size of kinetic events). It is important to underscore that the animals studied by Wolfe might have whisked in a different manner had they been engaged in a texture-discrimination task, but the fact that slip-stick kinetic signatures are invariant across experimental conditions suggests that they function as the fundamental input signal even during active texture discrimination.

**Figure 3:**
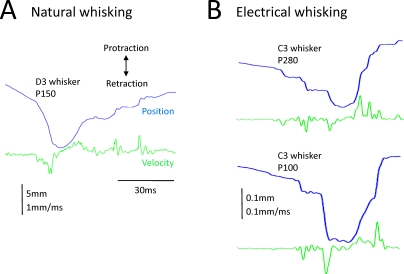
Comparison of Whisker Motion Profiles Collected under Different Conditions Kinetic signatures are apparent in position (blue traces) and velocity (green traces), and these are conserved across experimental conditions. (A) Contact of D3 whisker with texture P150, measured ~10 mm from the base (adapted from [12]). (B) Contact of C3 whisker with texture P280 (upper panel) and with texture P100 (lower panel), both measured 1 mm from the base (adapted from [10]).

**Figure 4:**
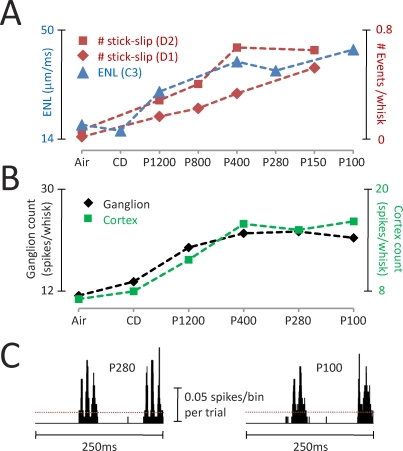
Candidate Texture-Coding Mechanisms (A) In two different studies, progressively coarser textures evoked kinetic signatures with higher magnitude kinetic events. Red marks are slip-stick events from [12] measured for whiskers D1 and D2; blue marks are ENL from [10] measured for whisker C3. (B) Higher ENLs led to higher firing rates in the trigeminal ganglion (black diamonds) and barrel cortex (green squares). Adapted from [10]. (C) Texture-specific spike patterns. Peristimulus time histogram (2 ms bins; 100 trials) of a ganglion cell for two whisks on textures P280 (left) and P100 (right). Mean firing rate (dashed red lines) were similar, suggesting temporal firing pattern as a critical coding mechanism.

When a whisker transmits a texture's kinetic signature to the sensory receptors, does the number of slip-stick events carry all the information, or does the temporal pattern of events carry additional information? To address this, we follow the sensory signal to its next processing step, the translation of motion profiles into neuronal activity. In anesthetized animals, neurons at all stages of the sensory pathway, from the trigeminal ganglion to barrel cortex, are effectively driven by high-speed and high-acceleration whisker movements [10,19–21]. Thus, texture-specific kinetic signatures obtained through electrical whisking are represented by differences in the overall rate of neuronal firing, which follow from the number and size of kinetic events. This “firing-rate coding” mechanism is illustrated in Figure 4B, where spike count per whisk, measured in the trigeminal ganglion and the barrel cortex (black diamonds and green squares, respectively), is given for the corresponding set of textures [10]. It is evident that the value of ENL for a given texture's motion profile was accurately translated to firing rate. Kinetic signatures are also represented by differences in the rhythm of neuronal firing, caused by the temporal pattern of kinetic events [10,22]. This “temporal pattern coding” mechanism is illustrated in Figure 4C, where electrical whisking on different grades of sandpaper—medium-grained (P280) and coarse-grained (P100)—evoked distinctive spike timing sequences in a ganglion cell [10].

From the P280 versus P100 example, we can see why the brain may have an advantage in using temporal pattern coding—it permits a much higher capacity for representing different stimuli [22]. On the other hand, trial-to-trial differences in the way whiskers engage a surface might cause temporal patterns of neural activity to vary, making this kind of code less robust than a firing rate code. The crucial test is in behaving animals; here, the evidence so far supports firing rate as a readout mechanism. In a simple rough versus smooth texture discrimination task, on correct trials, contacts with the rough texture evoked significantly higher firing rates in barrel cortex than did contact with the smooth texture [2]. On trials when the rat correctly identified the stimulus, the firing rate of neurons in barrel cortex was higher for rough than for smooth during a temporal window immediately preceding the instant of choice. This firing-rate code was reversed on error trials (lower for rough than for smooth). So the rat made its decision based upon the magnitude of whisker-evoked activity in barrel cortex. Although there is not yet any evidence pointing to the use of texture-specific firing patterns in behaving animals, it remains an intriguing possibility. Patterns could be essential whenever contact with the textures evokes nearly the same firing rate [22]. Just as rats shift their whisking strategy according to the textures they must discriminate [23], so might they adapt their strategy for decoding neuronal activity.

Since neurophysiologists and anatomists began to focus on the rodent whisker system in the 1970s, great strides have been made in unraveling the circuitry of the neuronal pathways that transmit information from the whiskers to the sensory cortex [24–26]. With the knowledge now available, this system provides an ideal opportunity for studying the connection between sensory receptors, neuronal activity, and perception. The kinetic signatures characterized by Wolfe and colleagues are a good building block for understanding texture sensation in the living animal.

## Abbreviations

ENL: equivalent noise level
